# A Systematic Review of Health Management Mobile Applications in COVID-19 Pandemic: Features, Advantages, and Disadvantages

**DOI:** 10.1155/2024/8814869

**Published:** 2024-01-09

**Authors:** Ali Garavand, Fatemeh Ameri, Fatemeh Salehi, Ali Hajipour Talebi, Zahra Karbasi, Azam Sabahi

**Affiliations:** ^1^Health Information Management, Department of Health Information Technology, School of Allied Medical Sciences, Lorestan University of Medical Sciences, Khorramabad, Iran; ^2^Health Information Technology, Student Research Committee, Department of Health Information Technology, School of Paramedical Sciences, Mashhad University of Medical Sciences, Mashhad, Iran; ^3^Health Information Management, Emam Reza Hospital, Mashhad University of Medical Sciences, Mashhad, Iran; ^4^Health Information Technology Expert, AJA University of Medical Sciences, Tehran, Iran; ^5^Health Information Management, School of Management and Medical Informatics, Kerman University of Medical Sciences, Kerman, Iran; ^6^Health Information Management, Department of Health Information Technology, Ferdows School of Health and Allied Medical Sciences, Birjand University of Medical Sciences, Birjand, Iran

## Abstract

**Introduction:**

With the increasing accessibility of smartphones, their use has been considered in healthcare services. Mobile applications have played a pivotal role in providing health services during COVID-19. This study is aimed at identifying the features, advantages, and disadvantages of health management mobile applications during COVID-19.

**Methods:**

This systematic review was conducted in PubMed, Scopus, and Web of Science using the related keywords up to November 2021. The original articles in English about the health management mobile applications in COVID-19 were selected. The study selection was done by two researchers independently according to inclusion and exclusion criteria. Data extraction was done using a data extraction form, and the results were summarized and reported in related tables and figures.

**Results:**

Finally, 12 articles were included based on the criteria. The benefits of mobile health applications for health management during COVID-19 were in four themes and 19 subthemes, and the most advantages of the application were in disease management and the possibility of recording information by users, digital tracking of calls, and data confidentiality. Furthermore, the disadvantages of them have been presented in two themes and 14 subthemes. The most common disadvantages are reduced adherence to daily symptom reports, personal interpretation of questions, and result bias.

**Conclusion:**

The study results showed that mobile applications have been effective in controlling the prevalence of COVID-19 by identifying virus-infested environments, identifying and monitoring infected people, controlling social distancing, and maintaining quarantine. It is suggested that usability, ethical and security considerations, protection of personal information, and privacy of users be considered in application design and development.

## 1. Introduction

Since the outbreak of the coronavirus in December 2019, the cases of COVID-19 in the world have been increasing rapidly. By August 21, 2020, over 226 million cases had been confirmed in the world, with more than 7.9 million deaths. Nonetheless, the recovery rates differ drastically in various countries due to different factors such as health interventions, effective planning, and outbreak management approaches [1, 2].

Due to the limitations of face-to-face clinical consultations, the healthcare systems face some challenges in delivering patient care. This experience underscored the inability of traditional methods alone to effectively manage the disease, highlighting the need for innovative solutions. In response, new information technologies, such as telemedicine and smartphones, were swiftly developed to enable optimal control of the virus [3]. The ability of society and hospitals to effectively manage and respond to widespread outbreaks of pandemics like COVID-19 can be enhanced. They could update technologies, which help governments identify, track, monitor, and treat patients with COVID-19 and manage human resources, economic resources, and time [4–7].

One of the key applications for coping with COVID-19 is the use of mHealth [8]. The World Health Organization's Global Observatory defines “mobile health (mHealth) as a public health practice supported by mobile devices such as mobile phones, patient monitoring devices, personal digital assistants, and other wireless devices” [9].

Nowadays, mHealth is used by all age groups, such as mother-child groups and older people [10, 11]. Populations in low- to middle-income areas benefit from mHealth applications through the provision of effective healthcare services, and governments that implement mHealth developed in low-income areas indirectly promote equity in access to healthcare services by bridging gaps in limited resources [12, 13].

Studies have revealed that mHealth educational applications and games have increased during quarantines [14–16]. Given the expansion of online systems, particularly in advanced countries, the intelligent use of existing data to identify future trends and current events or to identify comprehensive risks is considered a common and logical issue. When technologies focus on health, they provide tools that help communicate, structure, and organize data and information [17].

During COVID-19, emerging mobile applications could become a useful tool to help individuals with self-care, information, and crisis management and be a resource for health professionals to use [18]. Evidence indicates that mHealth has been used to empower healthcare providers to reach vulnerable people, conduct monitoring, and provide treatment, health-related education, and counseling [19–22]. Particularly, in the rapidly evolving environment of COVID-19, mHealth applications have hada key role in reducing cases of COVID-19 [3]. Additionally, mHealth applications are extensively used for various purposes to reduce the COVID-19 pandemic, such as self-assessment, contact tracing, information dissemination, minimizing exposure to the disease, reducing face-to-face visits, controlling disease symptoms, and facilitating access to healthcare [9, 23].

Echeverría et al.'s results on remote health monitoring in the workplace for early detection of COVID-19 cases using a mobile health app showed that the COVIDApp helps in early detection and rapid activation of protocols in the workplace, thus limiting the risk of spreading the virus and reducing the economic effect of COVID-19 on the manufacturing sector. The platform indicates the progress of the infection in real time and could assist in the design of new strategies [24]. Furthermore, a review study associated with the monitoring of symptoms of COVID-19 using mobile health applications indicated that the mHealth applications designed to monitor the symptoms of COVID-19 were of high quality and suggested that the program developers should follow the updated technical and clinical guidelines of the Centers for Disease Control and the World Health Organization (WHO) to better monitor the symptoms of COVID-19 [25]. Islam et al.'s results for exploratory analysis of mobile health applications developed for COVID-19 revealed that the key design features of mobile health applications are reliability, performance, usefulness, support, security, privacy, flexibility, responsiveness, ease of use, and cultural sensitivity [26].

However, there have been some studies about using mobile applications in health management. Initial reviews by the authors indicated that no systematic reviews have been done so far focusing on the capabilities, key functions, and challenges faced by mHealth programs in health management in the era of COVID-19. Therefore, given the capabilities that mobile health applications have in enhancing the process of self-care, communication, and crisis management and, on the other hand, identifying the features, advantages, and disadvantages of mobile health applications that developers can use in designing and developing them as best as possible, the study was conducted to review the developed mobile apps for health management in the COVID-19 era to identify the advantages, disadvantages, and the technologies used in these programs.

## 2. Material and Methods

### 2.1. Study Design and Search Strategy

This systematic review was conducted according to the PRISMA, which included the initial search, screening of studies, assessing the eligibility and risk of bias, and study selection [27]. The searches were done using a combination of keywords until 2021 (Table 1).

### 2.2. Data Sources

To find the studies, scientific databases, including PubMed, Scopus, and Web of Sciences, were searched according to the search strategy, and the found articles were imported into the EndNote reference manager software.

Additionally, to retrieve other related studies, a hand search was made in the list of references of the included articles and the Google Scholar search engine with different combinations of keywords.

### 2.3. Study Selection

The selection criteria comprised of original articles in English that introduced or reviewed health applications during the COVID-19 pandemic, specifically examining the advantages and disadvantages of these applications. This study excluded short articles, letters to the editor, conference abstracts, observational studies, review articles, and articles that were not available in full or written in a language other than English.

### 2.4. Data Extraction

The process of data extraction involved utilizing a designated data extraction form, based on the objectives of the study after selecting the studies. This form consisted of bibliographic information of articles, such as the first author's name, publication year, country of origin, and specifics regarding mobile health applications, such as the name of the application's name, the advantages and disadvantages, the technologies incorporated, the main outcome, and other outcomes. A content analysis of the studies was conducted following the study's objectives. The results of the analyses are given in tables and figures.

### 2.5. Quality and Risk of Bias Assessment

Quality assessment of studies was done by the Newcastle-Ottawa Scale (NOS), a widely recognized tool for assessing the quality of observational studies [28]. To accommodate the assessment of health information system quality in studies, some modifications were made to NOS [29]. All articles had the necessary quality according to the checklist.

## 3. Results

**Figure 1:**
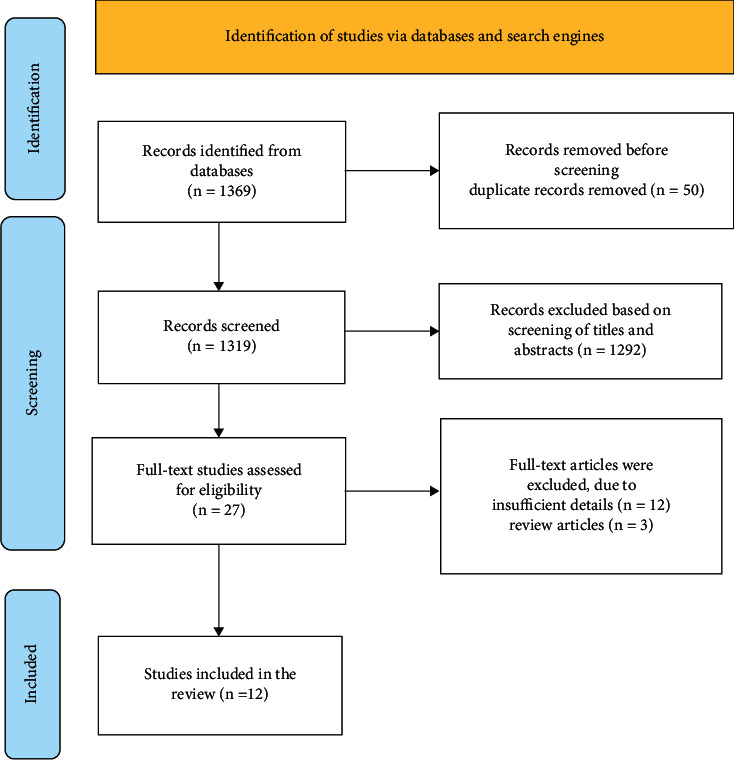
Study selection process.

In the initial review of three databases, 1369 articles were retrieved.  Figure 1 shows the study selection strategy process.

### 3.1. The Selected Studies

Table 2 represents the main characteristics of the included studies such as the country, type of technologies, and main outcomes.

According to the results, 58.33% of the studies were conducted in 2020 and 41.66% in 2021. The studies were conducted in the United States, Spain, England, Japan, Germany, the Netherlands, France, and Romania.

**Figure 2:**
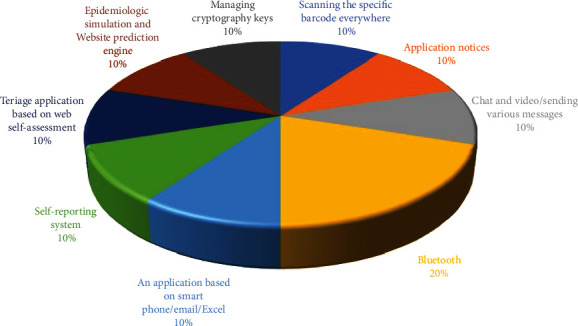
Technologies used in the reviewed studies.

Different technologies were used in the applications reviewed in the articles, and the details of these technologies are given in Figure 2.

### 3.2. Advantages of Mobile Health Applications

Table 3 presents the advantages of mobile health applications for health management during COVID-19 in four themes and 19 subthemes.

Table 3 indicates that mHealth applications had the most advantages in disease management, such as the possibility of recording information by users, digital tracking of calls, and data confidentiality.

### 3.3. Disadvantages of Mobile Health Applications

Table 4 presents the disadvantages of mobile health applications for health management during COVID-19 in healthcare institutions, organized into two themes and 14 subthemes.

Table 4 indicates the mHealth application's disadvantages in health management during the COVID-19 era, most of which are associated with reduced adherence to daily symptom reporting, personal interpretation of questions, and result bias.

## 4. Discussion

Quarantine during the COVID-19 pandemic significantly impacted people with respiratory diseases, because they faced challenges in accessing health services as easily as before [42]. Moreover, restrictions imposed by governments and advice to stay at home caused interventions and other healthcare services to be transferred to the online environment or various mobile platforms. This has created a significant opportunity for the effective use of mobile health applications during COVID-19. Moreover, mHealth applications in the era of COVID-19 are expanding due to their very important role in control, diagnosis, remote monitoring of disease symptoms, patient education, self-management of the disease, sharing data with healthcare professionals, and reducing hospital burden [43–45].

The study findings indicated that most of the apps designed with the general goal of helping to prevent the spread of the virus such as the use of masks, compliance with hygiene measures, and social distancing have been implemented. These findings are in line with other related studies [44].

According to the results, some of the most important technologies used in these applications included short messaging, telephonic and web-based programs, video chat, Bluetooth, and virtual reality and warning systems, in line with the findings of this study. Bluetooth technologies are used more often due to their low amounts of energy consumption and their effectiveness for contact tracing and mHealth applications [46].

It is suggested to use innovative technologies such as artificial intelligence (AI), machine learning (ML), and deep learning (DL) to enhance various approaches associated with the management of COVID-19, as AI, ML, and DL could prove effective in improving various approaches such as treatment, medication, screening, prognostication, contact tracing, and drug/vaccine development associated with healthcare services and management of COVID-19 [47, 48].

Considering the prevalent use of smartphones in society, one can leverage this technology to effectively manage similar situations. Hence, it is recommended to use the successful experiences in similar situations in the future.

Other results revealed that the applications designed for health management in the COVID-19 era have various advantages, such as identification of contaminated places based on barcodes, self-reporting of risk factors, sending reminders, early identification and isolation of people, examination of disease symptoms, observing health information according to the patient's health records, and providing self-assessment and disease warnings to their users. According to the results of the study, other studies include capabilities such as vehicle movement permits, providing prevention instructions, viewing the results of COVID-19 tests, supporting contact with the treatment staff, identifying health facilities and health in the surrounding areas, reporting suspicious cases, and booking clinic appointments and testing for COVID-19 [14].

According to Schmeelk et al.'s research, mHealth applications for monitoring the symptoms of COVID-19 are guiding users, the ability to communicate between patients and healthcare providers, and gathering demographic information of users such as age, name, date of birth, ethnicity, and geographic location. Moreover, the research findings indicated that the US is at the forefront of developing top-notch mHealth applications for monitoring COVID-19 symptoms. Notably, state-specific programs had the most updated and detailed information, likely due to collaborative efforts between local, county, or state health departments. These special state apps listed the most common symptoms and provided information on the number of local cases of COVID-19 and research advances. The strengths of the US mHealth apps were the long-term storage of symptom assessments, high-quality aesthetics, comprehensive symptom lists, and evidence-based information aligned with the Centers for Disease Control and Prevention (CDC) [25].

Furthermore, the results of Alanzi's review revealed that the features and functions of mHealth apps in the COVID-19 era are classified into seven categories: app overview (price, ratings, Android, iOS, developer/owner, country, and status), health tools (user status-risk assessment, self-assessment, E-pass integration, test result reporting, online consultation, and contact tracing), learning options (personalized notes, educational resources, and COVID-19 information), communication tools (query resolution, appointments, social network, and notifications), app design (data visualization, program plan), networking tools (location mapping-GPS, connectivity with other devices), and safety and security options (alerts, data protection). The study suggested that an integrated mHealth app with most of the features and capabilities analyzed in the study be designed and developed for managing COVID-19 [49].

Reviewing studies, data from professional organizations' websites, and standards governing software development for health or medical devices are advised to overcome barriers to mobile health apps. Nonadherence to treatment was reported as one of the disadvantages of health management applications in the era of COVID-19 in the present study. It is necessary to pay attention to adherence to treatment and health guidelines in the design of these applications, as the cornerstone of the management of the disease is prevention with the help of adherence to health protocols [50, 51]. Overall, many factors have a role in improving patients' adherence and satisfaction with the use of health information technologies.

In studies reporting positive outcomes, various factors have been considered in adherence. These include the utilization of user-friendly tools and equipment and the incorporation of usability in the design and development of telemedicine services, making services attractive to patients and motivating them. All of these factors contribute to an enhanced level of adherence. The provision of self-care equipment and services, without considering their design and usage, is inadequate for ensuring adherence. Furthermore, effective patient and healthcare provider interaction is crucial for the success of telehealth interventions [52].

Healthcare professionals have a key role in assisting patients in enhancing their self-management abilities and adherence. If healthcare providers lack a positive attitude towards interventions delivered via mHealth technologies, it may create uncertainty among patients regarding the use of these technologies, consequently leading to nonadherence to the prescribed treatment plan [53]. Furthermore, the consistency of treatment programs and easy and timely access to appropriate specialists are effective factors in increasing adherence [54].

The study results revealed that one of the limitations of using health management apps during COVID-19 was the preservation of patient information and confidentiality. Although mHealth apps offer many advantages, they also present substantial security and privacy challenges that may lead to data breaches, carrying potential social, legal, and financial ramifications [55, 56].

Some apps designed for the management of COVID-19, such as TraceTogether [57], Immuni [58], COVID Watch [57, 59], and PathCheck [58, 60], update their privacy policies so that no personal information that can divulge individuals' identity is not collected. These apps use also clear policies on data usage and destruction.

It is suggested that people's privacy should be considered when developing apps for public health and facing crises such as COVID-19. Thus, measures such as the following should be taken: placing the app on secure platforms, providing continuous updates for the app, requesting strong passwords from the users, authenticating users, and assuring users of their privacy during registration.

Given the wide use of COVID-19 management apps, despite the limitations of some apps, it is possible to help policymakers in deciding how to respond to COVID-19 and perform the necessary programs in this regard. Legal challenges, such as users' privacy, are critical issues that must be considered when developing health information systems [61, 62]. The utilization of cutting-edge digital technologies, including the incorporation of applications for managing infectious diseases, proves highly effective as a potent tool in disease control and prevention efforts [63].

## 5. Limitations

One limitation of the study is the inability to access certain applications as they are published in languages other than English. In the fast-paced realm of COVID-19 studies, where different communities have diverse needs, quick research has been conducted, potentially impacting the quality of studies and software products in this field. Nonetheless, the researchers attempted to select studies that have the required quality by applying rigorous inclusion and exclusion criteria.

## 6. Conclusion

A comprehensive study of mHealth apps associated with COVID-19 could help app developers understand the shortcomings of current apps and contribute to the more successful development of future apps by integrating various capabilities, functions, and innovative technologies. Physicians could review different apps and recommend the most effective one to patients so that they can take better control of their health and adopt strategies to mitigate COVID-19 by increasing awareness through mHealth apps.

The results indicated that mobile apps have proven effective in controlling COVID-19 by identifying contaminated environments, identifying and monitoring infected people, controlling social distancing, and maintaining quarantine. The user-friendly design and usability of the app, as well as the existence of privacy laws, are necessary conditions for the development of mHealth applications. It is suggested that interests and concerns, ethical and security considerations, protection of personal information, and privacy of users are taken into account. Actions such as paying attention to usability in the design and development processes of the app, placing the app on secure platforms, making continuous updates, requesting strong passwords from users, authenticating users, and ensuring users of their privacy during registration should be carried out.

It suggests in future studies that the experience of users, including managers, healthcare providers, and people, in using apps in different areas of the health system during the COVID-19 period, should be studied phenomenologically, to use their results in possible future crises.

## Figures and Tables

**Table 1:** Search strategy in scientific databases.

Time limitation	23 October 2021
Language limitation	Only full text in English
#1	“Mobile Application^∗^” OR “Portable Electronic App^∗^” OR “Portable Electronic” OR “Electronic Apps” OR “Portable Software Apps” OR “Portable Software”
#2	“COVID-19” OR “Coronavirus Disease 19” OR “SARS CoV 2 Infection” OR “SARS Coronavirus 2 Infection” OR “Coronavirus Disease 2019”
Search	#1 AND #2

**Table 2:** Characteristics of the studies included.

Authors, year	Country	Application name	Type of technologies	The main outcome	Other outcomes
Scherr et al. [30]	United States unit	MyCOVIDKey	Scanning the specific barcode for any location	The MyCOVIDKey application was a useful tool for tracking COVID-19 contacts in the university environment and was (1) effective in accurately tracking contacts, (2) not requiring much time, and (3) not requiring much effort	Compared with apps that work with Bluetooth and Global Positioning System (GPS), it creates more trust in users, and most users introduced it as simple to use

Zens et al. [31]	Germany	COVID-19 Symptom Tracker	—	Tracking self-reported symptoms helps identify new symptoms of COVID-19 and predict specific symptoms. Clinical screening through this tool allows rapid identification of infections and cost-effective use of testing resources	—

van Dijk et al. [32]	Netherlands	COVID RADAR	Application notifications	The ability to predict COVID-19 hotspots/in-application reported symptoms and behavior correlates with in-app reporting of SARS-CoV-2 testing. The predictive potential of the COVID RADAR correlates with external validation of in-app reported SARS-CoV-2 positive tests followed by government-reported case counts as well	Having confirmed the link between symptoms, social distancing behavior, and the number of COVID-19 cases, the next steps include trying to predict emerging hotspots by combining data on symptoms and social distancing behavior to quantify the risk of COVID-19 cases. Such predictions can be used to help with COVID-19 policy

Echeverría et al. [24]	Spain	COVIDApp	Chat and video/send a variety of messages including treatment recommendations or protocols	Rapid diagnosis of suspected and confirmed cases/reducing the risk of virus spread	Reducing the number of suspected cases/reduction in the number of long-term care facilities that were considered high risk for COVID-19/reduction in the total number of deaths, especially deaths among suspected cases

Varsavsky et al. [33]	England	COVID Symptom Study app	—	Mobile technology could be used to provide real-time data on the national and local status of the pandemic, enabling policymakers to make informed decisions about the COVID-19 pandemic and act as an independent and complementary source for traditional tools for disease monitor	Rapid diagnosis of cases in areas where testing is less. Polymerase chain reaction (PCR) test results and this self-report program can complement each other

Wymant et al. [34]	England	National Health Service (NHS) COVID-19	Bluetooth	Reducing the spread of COVID-19	Reducing the death rate due to disease

Nakamoto et al. [35]	Japan	COCOA	Smartphone/Bluetooth/private messaging services such as e-mail	Reducing the rate of transmission of infection/increasing the response pace to the epidemic/reducing workload/reducing operational errors/reducing population mobility/the efficiency and speed of identifying infected people	Protecting user data privacy from infected people, vulnerable people, attackers, and government authorities

Yamamoto et al. [36]	Japan	K-note	Smartphone/e-mail/Excel-based application	Preventing the spread of infection/in-app greatly reduced the follow-up burden for people who had close contact with known cases of confirmed COVID-19 infection	The app has helped with early diagnosis of COVID-19 or voluntary home quarantine of people with suspected symptoms, and the use of this app can ease the reopening of school and corporate activities/provide statistics to local governments and national headquarters

Soriano et al. [37]	Spain	Hospital Epidemics Tracker (HEpiTracker)	Smartphone-based program/self-report nature	Monitoring COVID-19 and other infectious diseases among the hospital staff	—

Denis et al. [38]	France	http://Maladiecoronavirus.fr	Self-assessment web-based triage program	A proper device to predict the increase in prevalence, hospitalizations, and intensive care unit admissions during the COVID-19 pandemic	Accurately predict the reduction in hospitalization rates

Getz et al. [39]	United States	NMB-DASA	The website's epidemiology simulation and prediction engine are based on a modified SEIR (susceptible, exposed, infectious, and improved) formula called SCLAIV	Managing epidemics and reducing the level of economic damage from the COVID-19 pandemic/the availability of computational tools, such as http://covid-webapp.numerusinc.com, allows policymakers and healthcare managers to predict the onset of an outbreak. Next, get into trouble less than what was the case with COVID-19	Helping policymakers decide how to respond to the novel coronavirus

Stanciu et al. [40]	Romania	TAMEC	(i) Management of cryptographic keys to ensure the confidentiality of system data and computing server (CS) web service (ii) Client configuration web service (iii) DSP-WebApp program (iv) TAMEC map system (v) Graphical web interface	Facilitating the prevention of the spread of the SARS-CoV-2 virus in the community	To help limit the spread of disease in the population and reduce epidemiological studies of healthcare workers

**Table 3:** The advantages of health management mobile applications.

Main groups	Subgroups
The possibility of registering and self-evaluating by the user	(i) The possibility of entering information associated with risk factors, symptoms of COVID-19, health status, and COVID-19 status by the user [31, 32, 36, 38, 39] (ii) The possibility of frequent self-assessment of health status [30, 38]

Providing feedback to the user	(i) Providing personal advice to the user according to its location [30] (a) Sending a daily reminder to the user [32] (b) Presenting appropriate local information about the prevention of COVID-19 [34] (c) Notification of the result of the COVID-19 test [34] (ii) Quarantine is recommended if symptoms of COVID-19 are observed, or see a doctor or call the emergency room if symptoms of shortness of breath and anorexia are reported [38] (iii) Responding to questions associated with social distancing, patient isolation, and vaccination [39] (iv) Presenting a quick warning if the user sees contact with a person infected with COVID-19 [40] (v) Presenting the prevalence and occurrence rate by user self-report data [33]

Identifying places and people infected with COVID-19	(i) Location scanning using a special barcode [30] (ii) Early identification and isolation of people suspected of COVID-19 [41] (iii) Identifying the main points of the disease [33] (iv) Examining places using a QR code scanner [34] (v) Digital tracking of cases and calls [34, 37, 40] (vi) Automatic recording of close calls (distance of one meter for 15 minutes) on mobile phone using Bluetooth technology [35]

Confidentiality and privacy	(i) The possibility of deleting information if requested by the user [32, 35] (ii) Obtaining the informed consent of a patient with COVID-19 for identity verification [35] (iii) It offers a high degree of confidentiality for the data of all users and protects their privacy [36, 40]

**Table 4:** Disadvantages of health management mobile applications.

Main groups	Subgroups
Problems with recording, storing, sharing, uploading, and analyzing data	(i) Not connecting consecutive user data [34] (ii) Impossibility of uploading data [39] (iii) Delay in data sharing [35] (iv) Not predicting the long-term effects of the disease [35] (v) Analysis in a limited way and at the national and state level [39] (vi) Not predicting the hospitalization period [38]

Problems with the method and technology used	(i) Lack of access to smartphones and Wi-Fi connections [36] (ii) Contact identification with Bluetooth and without using GPS [40] (iii) Inefficiency of e-mail for data exchange [36] (iv) Lack of strict monitoring and follow-up of users [38] (v) Concern over privacy [37] (vi) Not confirming the results and reports recorded by the user [31] (vii) Personal interpretation of questions and result bias [32, 33] (viii) Reluctance and reduced adherence to daily symptom reporting [37, 38]

## Data Availability

All data extracted and reported are included in this published article.
